# Systematic Review of Grief Interventions

**DOI:** 10.1093/geroni/igaa057.1354

**Published:** 2020-12-16

**Authors:** Nicole Ricken, Alice Kim, Mona Khaled, Geoff Corner, Christopher Beam

**Affiliations:** University of Southern California, Los Angeles, California, United States

## Abstract

Grief interventions address pain and suffering in response to the death of a significant other. Evidence-based grief psychotherapies treat normative grief to symptoms of persistent complex bereavement disorder, the latter of which is characterized by difficulty accepting the loss and persistent yearning for the decedent. We reviewed published randomized controlled trials (RCT) of grief-focused psychotherapies to test two hypotheses. First, participants receiving grief-focused psychotherapies should demonstrate decline of grief symptoms from pre- to post-intervention. Second, participants receiving grief-focused psychotherapies should demonstrate lower grief symptoms post-intervention than participants receiving control treatments. Published studies were identified using academic search engines (Web of Science, PsycInfo, and Google Scholar) and by reviewing reference sections of published RCTs. Twelve published RCTs were identified. Effect sizes (Hodges’ g) and confidence intervals were calculated. Results support our first hypothesis and partially support our second hypothesis. Grief-related symptoms declined from pre- to post-intervention (g ranged from -0.39 to -2.51), with all studies reporting statistically significant effects. When comparing post-intervention differences in grief-focused psychotherapies versus control groups, effects were more variable (g ranged from -2.40 to 3.02), with seven studies demonstrating greater improvement among grief intervention recipients than control treatment recipients. Grief interventions appear to be effective for reducing grief-related symptoms pre- to post-intervention. However, they were only more effective than control treatments in just over half of published RCTs. While grief interventions were more effective, point estimate ranges are wide, suggesting that treatment effectiveness probably depends on factors other than the treatments themselves.

